# Prolonged Sojourn at Very High Altitude Decreases Sea-Level Anaerobic Performance, Anaerobic Threshold, and Fat Mass

**DOI:** 10.3389/fphys.2021.743535

**Published:** 2021-10-05

**Authors:** Robert K. Szymczak, Tomasz Grzywacz, Ewa Ziemann, Magdalena Sawicka, Radosław Laskowski

**Affiliations:** ^1^Department of Emergency Medicine, Faculty of Health Sciences, Medical University of Gdańsk, Gdańsk, Poland; ^2^Department of Sport, Institute of Physical Culture, Kazimierz Wielki University in Bydgoszcz, Bydgoszcz, Poland; ^3^Department of Athletics, Strength and Conditioning, Poznań University of Physical Education, Poznan, Poland; ^4^Department of Neurology, Faculty of Medicine, Medical University of Gdańsk, Gdańsk, Poland; ^5^Department of Physiology and Biochemistry, Gdańsk University of Physical Education and Sport, Gdańsk, Poland

**Keywords:** altitude, chronic hypoxia, physical capacity, body composition, extremes, mountaineering

## Abstract

**Background:** The influence of high altitude on an organism’s physiology depends on the length and the level of hypoxic exposure it experiences. This study aimed to determine the effect of a prolonged sojourn at very high altitudes (above 3,500m) on subsequent sea-level physical performance, body weight, body composition, and hematological parameters.

**Materials and Methods:** Ten alpinists, nine males and one female, with a mean age of 27±4years, participated in the study. All had been on mountaineering expeditions to 7,000m peaks, where they spent 30±1days above 3,500m with their average sojourn at 4,900±60m. Their aerobic and anaerobic performance, body weight, body composition, and hematological parameters were examined at an altitude of 100m within 7days before the expeditions and 7days after they descended below 3,500m.

**Results:** We found a significant (*p*<0.01) decrease in maximal anaerobic power (MAP_WAnT_) from 9.9±1.3 to 9.2±1.3W·kg^−1^, total anaerobic work from 248.1±23.8 to 228.1±20.1J·kg^−1^, anaerobic threshold from 39.3±8.0 to 27.8±5.6 mlO_2_·kg^−1^·min^−1^, body fat mass from 14.0±3.1 to 11.5±3.3%, and a significant increase (*p*<0.05) in maximal tidal volume from 3.2 [3.0–3.2] to 3.5 [3.3–3.9] L after their sojourn at very high attitude. We found no significant changes in maximal aerobic power, maximal oxygen uptake, body weight, fat-free mass, total body water, hemoglobin, and hematocrit.

**Conclusion:** A month-long exposure to very high altitude led to impaired sea-level anaerobic performance and anaerobic threshold, increased maximal tidal volume, and depleted body fat mass, but had no effect on maximal aerobic power, maximal oxygen uptake, or hemoglobin and hematocrit levels.

## Introduction

Altitude acclimatization is defined as the sum of positive changes in an organism that reduce the risk of acute altitude illness and improve performance in hypoxic conditions at altitudes above 2,500m (West et al., 2007). The process of acclimatization focuses on increasing ventilation and raising hematocrit levels. These improvements occur within days to weeks at altitude (Lundby et al., 2004). Apart from the respiratory and hematological changes, acclimatization involves other systems that adjust to altitude over different periods, so mountaineers struggle to determine when they have been sufficiently acclimatized to high altitudes. Guidelines for mountaineers therefore include setting adequate ascent rates to higher altitudes, which are contingent on the highest altitude of sojourn being targeted (Kupper et al., 2012; Luks, 2012). Pollard and Murdoch (1997) and Broadhurst (2008) classify the range of altitudes that affect climbers as sea level (<1,500m), moderate (1,500–2,500m), high (2,500–3,500m), very high (3,500–5,500m), extreme (5,500–7,500m), and death zone (>7,500m). An ascent rate of 300–500m per day increases in sleeping elevation over 2,500m is recommended at high and very high altitudes (2,500–5,500m; Luks, 2012). Acclimatization profiles for expeditions to extreme altitudes (>5,500m) often involve “yo-yo tactics,” where ascents to high camps are separated by rest in the base camp, usually between 3,500 and 5,500m. Camps are usually 1,000m in altitude apart, so overnight stays are not recommended for the first ascent to higher camps (Kupper et al., 2012).

Prolonged exposure to altitude not only involves the “positive changes” of acclimatization, but also “negative changes” of high-altitude deterioration that affect physical and mental condition (Ward, 1954; West et al., 2007). These deleterious effects include impaired physical performance (Cerretelli and di Prampero, 1985; Hoppeler et al., 1990a; Cerretelli, 1992), recovery from fatigue (Milledge et al., 1977), disturbed sleep (Weil and White, 2001), cognitive disorders (Raichle and Hornbein, 2001), and weight loss (Boyer and Blume, 1984). Deterioration becomes evident after sojourns longer than 5weeks at extreme altitudes (>5,000–5,500m), which are commonly endured by climbers on 8,000m peak expeditions where the base camps are situated above 5,000m. Sea-level maximal oxygen uptake decreases after 6weeks above 5,200m with some exposure to altitudes above 8,000m (Hoppeler et al., 1990a), as does maximal anaerobic power (MAP_WAnT_) after 5weeks above 5,200 with ascents to higher altitudes (Cerretelli and di Prampero, 1985). Reductions in muscle mass, fiber cross-sectional area, and mitochondrial density in muscle fibers (Hoppeler et al., 2003) are the main reasons for the deterioration in physical performance after chronic hypoxia.

Typical expeditions to 6,000–7,000m peaks usually last 3–5weeks and site their base camps at 3,500–4,500m (Salisbury and Hawley, 2011). Our study aimed to determine the effect of a prolonged sojourn at very high altitudes (above 3,500m) on subsequent sea-level physical performance, body weight, body composition, and hematological parameters in alpinists who participated in expeditions to 7,000m peaks.

## Materials and Methods

### Materials

**Figure 1:**
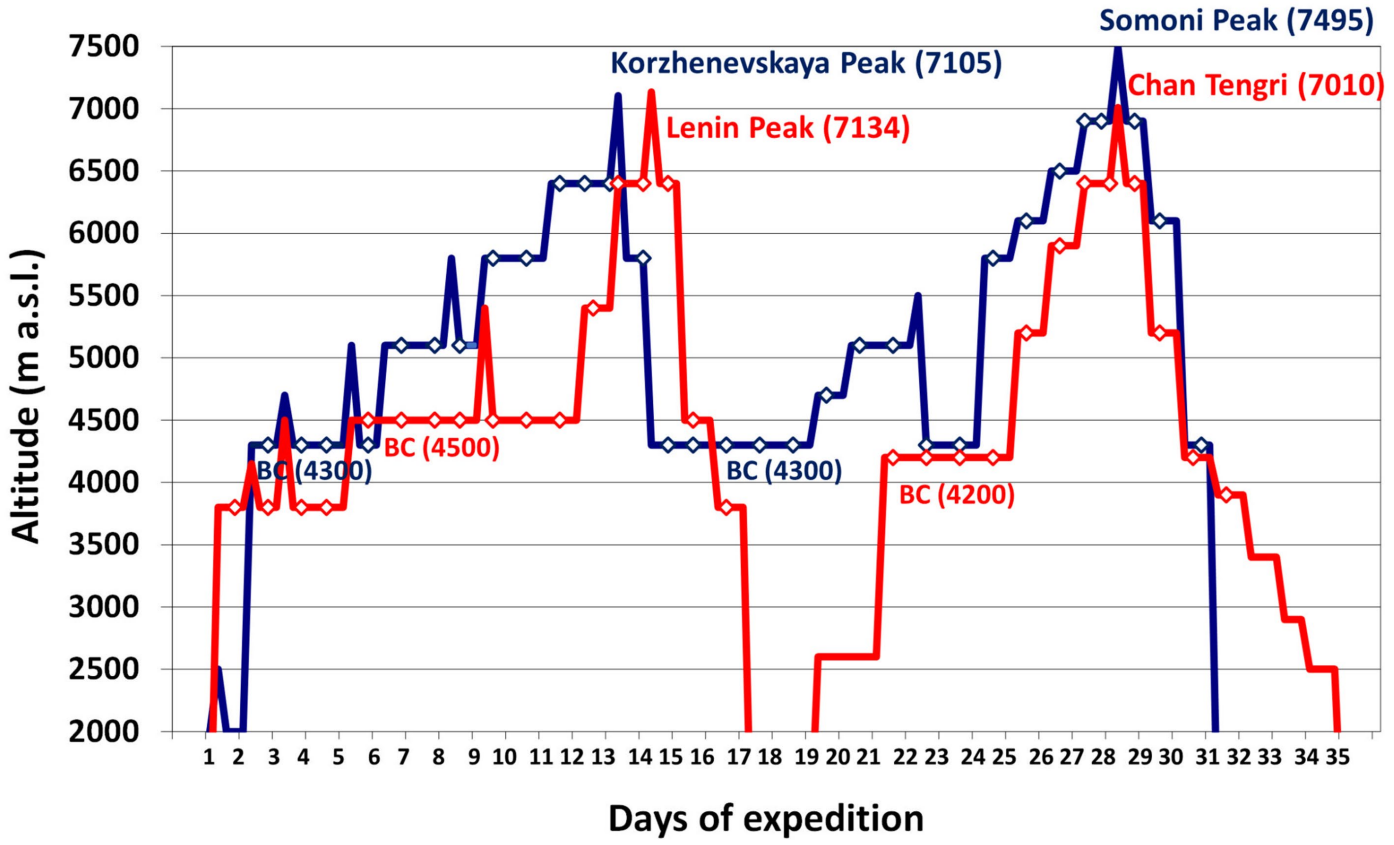
The rate of ascent of the expeditions we analyzed. Legend: red line – Kyrgyzstan expedition to Lenin Peak (7,134m) and Chan Tengri (7,010m); blue line – Tajikistan expedition to Korzhenevskaya Peak (7,105m) and Somoni Peak (7,495m); diamonds – sleeping altitude. BC, base camp.

Ten Caucasian mountaineers (nine males and one female) with a mean age of 27±4years (range 20–34) participated in the study. Five mountaineers climbed Lenin Peak (7,134m) and Chan Tengri (7,010m) in Kyrgyzstan, and the other five climbed Korzhenevskaya Peak (7,105m) and Somoni Peak (7,495m) in Tajikistan. They spent 30±1days above 3,500m at an average altitude of 4,900±60m. Both expeditions averaged 17±1 climbing days and 13 rest days. We based the average altitude of their sojourn on the altitudes at which the climbers slept (Figure 1). Both groups were analyzed together because of the similar character of their expeditions, their plans to climb two 7,000m peaks, the altitude of the base camps above 4,000m, and the duration of their sojourns. Each of the climbers agreed to participate in the study, which was approved by the Regional Ethics Committee of the Medical University of Gdańsk.

### Methods

We examined aerobic and anaerobic performance, body weight and composition, and hematological parameters at an altitude of 100m within 7days before each expedition and 7days after the participants had descended below 3,500m. We conducted body composition and hematological parameters measurements in the morning and on an empty stomach. On the same day, an hour after a light meal, we performed anaerobic test. The next day, in the morning, an hour after a light meal, we measured aerobic performance. All the tests were performed during the follicular phase of the female subject’s menstrual cycle.

#### Aerobic Performance

We measured aerobic performance in an incremental exercise test to exhaustion on an ER900 cycle ergometer (Jaeger-Viasys, Germany). Aerobic performance we assessed directly with an expiratory gas analyzer (Oxycon Pro, Jaeger-Viasys, Germany) and computer software (Breath by Breath). We used a protocol of Wasserman et al. (1987) for the measurements. The main stage of the measurement was an exercise till refuse, with an incremental load of 25W a minute, while continuously monitoring and recording oxygen uptake (VO_2_) and carbon dioxide excretion (VCO_2_), both in ml·kg^−1^·min^−1^ and L·min^−1^; respiratory quotient (RQ); minute ventilation (VE) as L·min^−1^; tidal volume (VT) in L; breath frequency (BF) as breaths·min^−1^; heart rate (HR) as beats·min^−1^; and aerobic power (MP_VO2_) in W, W·kg^−1^ and W·kgFFM^−1^.

We measured maximal oxygen uptake (VO_2_max) directly and determined anaerobic threshold (AT) using a ventilation method. The ventilation method determined anaerobic threshold when the increase in minute ventilation rose disproportionately to the increased load (Wasserman et al., 1973; Yoshida et al., 1987). This threshold has also been defined using the respiratory exchange ratio (RER; Solberg et al., 2005): RER≥1 means an increase in carbon dioxide excretion, which is an indirect sign of increased anaerobic glycolysis. We therefore expressed anaerobic threshold in mlO_2_·kg^−1^·min^−1^, percent of maximal oxygen uptake (%VO_2_max), beats·min^−1^, percent of maximal heart rate (%HRmax), W, and percent of maximal aerobic power (%MP_VO2max_).

#### Anaerobic Performance

We measured anaerobic performance during a supramaximal cycloergometric exercise for 30s in the Wingate Anaerobic Test (WAnT; Dotan and Bar-Or, 1983; Bar-Or, 1987; Inbar et al., 1996). The test was performed on an Ergomedic E818 cycloergometer (Monark, Sweden). The load on the flywheel was set at the beginning of the exercise and was determined individually for each participant at the level of 0.075kG per kilogram of body weight. We used MCE v 2.0 computer software to calculate the parameters we measured in the WAnT (Berry et al., 1989). The parameters we assessed were total anaerobic work (Wtot) in kJ, J·kg^−1^ and J·kgFFM^−1^; MAP_WAnT_ in W, W·kg^−1^, and W·kgFFM^−1^; time to reach maximal power (TR_MAP_), and time of maintaining the maximal power (TM_MAP_) in seconds (s); and the power drop index (PDI) as a percentage. The proportion between the phosphagenic (Wana_phosph_) and the glycolytic (Wana_glycol_) component of total anaerobic work was expressed as a percentage of total anaerobic work (%Wtot) in the WAnT (Serresse et al., 1988; Ziemann et al., 2011).

#### Anthropometric Measurements

Body weight (BW) and body composition were estimated using a bioelectrical impedance floor scale (TBF-300 Body Fat Monitor/Scale Analyzer, Tanita, Japan), assessing body weight (BW), fat-free mass (FFM), and total body water (TBW) in kg; fat mass (FAT) in kg and as a percentage; body mass index (BMI) in kg·m^−2^; body surface area (BSA) in m^2^; and basic metabolic rate (BMR) in kcal.

#### Hematological Measurements

We express red blood cell (RBC) counts in 10^12^/L, hematocrit (Hct) as a percentage, and blood hemoglobin concentration (Hb) as g/dl in venous blood samples collected from superficial veins of participants’ upper limbs. Measurements were made with the COULTER® LH 750 Hematology Analyzer (Beckman-Coulter, United States).

### Statistical Analysis

We used Statistica 13.1 (StatSoft, United States) for our statistical analyses, testing the normality of data with the Shapiro-Wilks W test, then the homogeneity of variance with the Brown-Forsythe test.

The differences between the results of physical performance, body composition, and blood parameters at sea level before and after the expeditions were analyzed using Student’s *t* test (T) for parametric variables and Wilcoxon’s rank test (W) for nonparametric variables.

We also calculated the effect size (partial eta^2^), ranging between 0 and 1. Using Cohen’s rule of thumb and the conversion table for eta^2^, the interpretations of the partial eta^2^ value are unequivocal. The most restricted interpretation method assigns values of partial eta^2^ to the effect size as 0.1 for a small effect, 0.3 for a medium effect, and 0.5 for a large effect (Cohen, 1988; Nakagawa and Cuthill, 2007; Fritz et al., 2012).

Our results are expressed as means with a standard deviation (*M*±*SD*) for parametric variables and as medians (MD) with a lower quartile (LQ) and an upper quartile (HQ) for nonparametric variables. We set the statistical significance at *p*<0.05 for all our analyses.

## Results

### Aerobic Performance

Maximal tidal volume increased from 3.2L before a period of chronic hypoxia to 3.5L afterwards (*p*<0.05, eta^2^=0.37; Table 1). We noted an average 29% decrease in all values for anaerobic threshold after a month at very high altitude (*p*<0.01, eta^2^=0.38–0.49; Table 1), but we observed no significant decrease in maximal aerobic power, maximal heart rate, or maximal oxygen uptake after the period of chronic hypoxia. Maximal minute ventilation did not increase significantly and maximal breath frequency showed no changes (Table 1).

**Table 1:** Parameters of aerobic performance measured before and after 1month at very high altitude (mean altitude of 4,900±60m).

Aerobic performance parameters	Before chronic hypoxia M (±SD) or MD (LQ-HQ)		After chronic hypoxia M (±SD) or MD (LQ-HQ)		Test	*p*	Effect size
MP_VO2max_ [W]	359.0	(±57.3)	338.0	(±71.5)	T	0.12	0.09
MP_VO2max_ [W·kg^−1^]	4.9	(±0.6)	4.7	(±0.6)	T	0.17	0.08
MP_VO2max_ [W·kgFFM^−1^]	5.8	(±0.6)	5.4	(±0.7)	T	0.07	0.18
HRmax [beats·min^−1^]	187	(±10)	183	(±13)	T	0.08	0.19
VEmax [L·min^−1^]	143.5	(±39.1)	158.2	(±37.7)	T	0.18	0.06
VTmax [L]	3.2	(3.0–3.2)	3.5	(3.3–3.9)	W	0.01	0.37
BFmax [breath·min^−1^]	53.4	(±12.6)	53.1	(±8.1)	T	0.93	0.002
VO_2_max [L·min^−1^]	3.7	(±0.9)	3.3	(±0.8)	T	0.24	0.06
VO_2_max [ml·kg^−1^·min^−1^]	50.1	(±8.2)	46.8	(±7.8)	T	0.35	0.05
AT [mlO_2_ kg^−1^·min ^1^]	39.3	(±8.0)	27.8	(±5.6)	T	0.002	0.39
AT [%VO_2max_]	78.5	(±10.3)	59.8	(±9.2)	T	0.001	0.41
AT-HR [beat·min^−1^]	163	(±17)	132	(±13)	T	0.001	0.43
AT [%HR_max_]	87.2	(±7.4)	71.9	(±5.0)	T	0.001	0.38
AT [W]	275	(215–300)	150	(125–200)	W	0.005	0.49
AT [%MP_VO2max_]	73.7	(±10.7)	48.4	(±8.9)	T	0.001	0.44

Data as means (M)±SD for parametric distribution and medians (MD) with lower quartile (LQ) and upper quartile (HQ) for nonparametric variables. Statistical tests: *t* test (T) for variables with parametric distribution; Wilcoxon rank test (W) for variables with nonparametric distribution. Effect size expressed as partial eta^2^; maximal aerobic power (MP_VO2max_); maximal heart rate (HRmax); maximal minute ventilation (VEmax); maximal tidal volume (VTmax); maximal breath frequency (BFmax); maximal oxygen uptake (VO_2_max); and anaerobic threshold (AT).

### Anaerobic Performance

Maximal anaerobic power decreased significantly from 721.7W before the expedition to 643.3W after the period of hypoxia (*p*<0.001, eta^2^=0.38). Maximal anaerobic power also decreased significantly in relative values from 9.9 to 9.2W·kg^−1^ (*p*<0.01, eta^2^=0.33) and from 10.9 to 9.6W·kgFFM^−1^ (*p*<0.1, eta^2^=0.28; Table 2). Our analysis of total anaerobic work showed a significant decrease from 18.0kJ measured before the expedition to 15.9kJ after the sojourn at high altitude (*p*<0.001, eta^2^=0.42). Total anaerobic work decreased significantly in relative values, from 248.1 to 228.1J·kg^−1^ (*p*<0.01) and from 291.4 to 261.7J·kgFFM^−1^ (*p*<0.001, eta^2^=0.41; Table 2).

**Table 2:** Anaerobic performance parameters measured before and after 1month at very high altitude (mean altitude of 4,900±60m).

Anaerobic performance parameters	Before chronic hypoxia M (±SD) or MD (LQ-HQ)		After chronic hypoxia M (±SD) or MD (LQ-HQ)		Test	*p*	Effect size
MAP_WAnT_ [W]	721.7	(±159.2)	643.3	(±162.3)	T	0.001	0.38
MAP_WAnT_ [W·kg^−1^]	9.9	(±1.3)	9.2	(±1.3)	T	0.003	0.33
MAP_WAnT_ [W·kgFFM^−1^]	10.9	(10.7–13.0)	9.6	(9.4–11.9)	W	0.02	0.28
TR_MAP_ [s]	4.2	(3.6–4.4)	4.4	(2.6–4.9)	W	0.7	0.12
TM_MAP_ [s]	4.4	(±1.6)	3.9	(±1.1)	T	0.3	0.14
PDI [%]	19.0	(±4.3)	18.3	(±5.5)	T	0.7	0.09
Wtot [kJ]	18.0	(±3.4)	15.9	(±3.2)	T	0.0003	0.42
Wtot [J·kg^−1^]	248.1	(±23.8)	228.1	(±20.1)	T	0.002	0.33
Wtot [J·kgFFM^−1^]	291.4	(±22.5)	261.7	(±20.5)	T	0.0003	0.41
Wana_phosph_ [%W_tot_]	38.6	(±10.2)	34.9	(±11.2)	T	0.6	0.11
Wana_glycol_ [%W_tot_]	83.9	(±12.1)	77.0	(±13.4)	T	0.1	0.17

Data as means (M)±SD for parametric distribution, medians (MD) with lower quartile (LQ) and upper quartile (HQ) for nonparametric distribution of variables; Statistical tests: *t* test (T) for variables with parametric distribution; Wilcoxon rank test (W) for variables with nonparametric distribution. Effect size expressed as partial eta^2^; Maximal anaerobic power (MAP_WAnT_); time to reach maximal power (TR_MAP_); time of maintaining maximal power (TM_MAP_); power drop index (PDI); total work (Wtot); and phosphagenic (Wana_phosph_) and glycolytic (Wana_glycol_) component of total anaerobic work.

We observed no difference before and after the period of chronic hypoxia in the time to reach maximal anaerobic power or in the time of maintaining maximal anaerobic power, in the PDI, or in the proportion of phosphagenic and glycolytic components of the total anaerobic work (Table 2).

### Anthropometric Measurements

Chronic hypoxia induced a significant decrease in fat mass, from an average of 10.2kg before the expedition to 8.1kg after a month at very high altitude (*p*<0.001, eta^2^=0.47). Similarly, the percentage of fat mass decreased significantly from 14% before the expedition to 11.5% afterwards (*p*<0.001, eta^2^=0.44; Table 3). Fat mass in kg and % decreased in all participants. Analysis of body weight, fat-free mass, total body water, body mass index, body surface area, and basic metabolic rate did not show any significant changes after the period of chronic hypoxia.

**Table 3:** Anthropometric parameters measured before and after 1month at very high altitude (mean altitude of 4,900±60m).

Anthropometric parameters	Before chronic hypoxia M (±SD) or MD (LQ-HQ)		After chronic hypoxia M (±SD) or MD (LQ-HQ)		Test	*p*	Effect size
BW [kg]	76.9	(65.5–77.3)	71.9	(67.8–78.3)	W	0.16	0.19
FAT [%]	14	(±3.1)	11.5	(±3.3)	T	0.0001	0.44
FAT [kg]	10.2	(±2.3)	8.1	(±1.8)	T	0.0001	0.47
FFM [kg]	65.8	(59.6–66.9)	63.3	(59.2–69.0)	W	0.9	0.006
TBW [kg]	48.2	(43.6–49.0)	46.3	(43.3–50.5)	W	0.9	0.007
BMI [kg·m^−2^]	22.8	(±2.1)	22.3	(±1.8)	T	0.16	0.09
BMR [kcal]	1,825.5	(1,678–1,883)	1,758.5	(1,656–1,881)	W	0.16	0.14
BSA [m^2^]	1.9	(±0.1)	1.9	(±0.1)	T	0.16	0.0001

Data are expressed as means (M)±SD for parametric distribution, medians (MD) with lower quartile (LQ) and upper quartile (HQ) for nonparametric distribution of variables; Statistical tests: t test (T) for variables with parametric distribution; Wilcoxon rank test (W) for variables with nonparametric distribution. Effect size expressed as partial eta^2^; Body weight (BW); fat mass (FAT); fat-free mass (FFM); total body water (TBW); body mass index (BMI); basic metabolic rate (BMR); and body surface area (BSA).

### Hematological Measurements

Our analysis of blood parameters did not show any significant changes after the period of chronic hypoxia (Table 4).

**Table 4:** Blood parameters measured before and after 1month of sojourn at a very high altitude (mean altitude of 4,900±60m).

Blood parameters	Before chronic hypoxia M (±SD) or MD (LQ-HQ)		After chronic hypoxia M (±SD) or MD (LQ-HQ)		Test	*P*	Effect size
RBC [10^12^/L]	5.3	(4.8–5.4)	5.3	(4.9–5.4)	W	0.78	0.0003
Hct [%]	47.2	(45.0–47.8)	47.6	(43.7–49.0)	W	0.57	0.002
Hb [g/dl]	15.0	(±1.5)	15.0	(±1.1)	T	0.95	0.0001

Data are expressed as means (M)±SD for parametric distribution, medians (MD) with lower quartile (LQ) and upper quartile (HQ) for nonparametric variables. Statistical tests: *t* test (T) for variables with parametric distribution, Wilcoxon rank test (W) for variables with nonparametric distribution. Effect size expressed as partial eta^2^; red blood cell counts (RBC), hematocrit (Hct), and blood hemoglobin concentration (Hb).

## Discussion

### Dose of Hypoxia

We struggled to compare our results with those of certain authors because of the imprecisely defined methods of calculating a dose of hypoxia. The period of exposure and the level of hypoxia are different in almost all studies. The dose of hypoxia mountaineers experience on extreme-altitude expeditions to 8,000m peaks is usually presented in days above the base camp, ignoring the altitude of the higher camps in which the alpinists spend considerable time (Boyer and Blume, 1984; Cerretelli and di Prampero, 1985; Ferretti et al., 1990b; Hoppeler et al., 1990a; Cerretelli, 1992; Reynolds et al., 1999; Mizuno et al., 2008; Doria et al., 2020). By our calculations, mountaineers on typical Mount Everest climbing itineraries spend 42days (6weeks) at or above the base camp (>5,300m). Acclimatization plans usually include three rotations to higher camps before the summit bid and within the 42days these mountaineers usually spend an equal number days of climbing and resting at an average altitude of about 6,000m (Boukreev and DeWalt, 2002). The new metrics of hypoxic dose based on elevation and duration of exposure “kilometer hours” (Garvican-Lewis et al., 2016) or sustained duration at a given arterial saturation “saturation hours” (Millet et al., 2016) were proposed. According to a model presented by Garvican-Lewis et al. (2016), the hypoxic dose experienced by climbers in our study was ~3,500km∙h, while on typical Mount Everest expedition alpinists are exposed to the hypoxic dose of approximately 6,000km∙h. Unfortunately, the model is not widely used.

The effects of similar doses of hypoxia might differ in natural and simulated conditions. Experiments performed in simulated conditions allow researchers to control many confounding variables and enable them to focus on the effects of hypoxia on the human organism (Rose et al., 1988; Green at el., 1989; Westerterp-Plantenga et al., 1999). Mountaineering expeditions, however, experience many variables – such as low temperature, high wind, low humidity, high UV radiation, dietary restrictions, psychological stress, and great physical effort – apart from hypobaric hypoxia (Szymczak et al., 2021a,b). These confounding variables likely affect the results of these studies (Ferretti et al., 1990b; Hoppeler et al., 1990a; Reynolds et al., 1999; Mizuno et al., 2008). Authors also seldom describe the mountaineers’ climbing style and do not provide data concerning climbing intensity. The dose of hypoxia is probably different in expedition-style mountaineering and in alpine-style expeditions. In expedition-style mountaineering, the climbers leave base camp multiple times to establish higher camps and then return to base camp; alpine-style expeditions climb the mountain in a single push. Comparative studies analyzing members of mountaineering expeditions should also recognize that climbers’ physical performance varies widely (Hoppeler et al., 1990a; Garrido et al., 1997). The dose of hypoxia climbers experience should be precisely defined to aid comparing different studies. Giving a dose of hypoxia in “kilometer hours” (Garvican-Lewis et al., 2016) or “saturation hours” (Millet et al., 2016) would greatly improve the comparability of the results of different studies.

### Aerobic Performance

Hypoxia-inducible factor 1 (HIF-1) plays a key role in humans’ adaptation to inadequate oxygen supply by stimulating erythropoiesis (Semenza, 2000), thus increasing arterial oxygen content. The HIF-1 response to hypoxia is time-dependent and down-modulated due to acclimatization (Lundby et al., 2009). Our study found no changes in the hematological parameters we measured, which might account for the unchanged aerobic performance we observed. The lung’s oxygen diffusive capacity increases in hypoxic conditions, which might improve aerobic performance. This improved oxygen diffusive capacity might result from the greater number of lung capillaries that develop after prolonged exposure to hypoxic conditions (Howell et al., 2003) or because more existing capillaries are recruited during acclimatization (Capen and Wagner, 1982). The level of improved oxygen diffusive capacity seems to depend on the dose of hypoxia. Lundby et al. (2004) observed that a 2-week sojourn at 4,100m does not increase the oxygen diffusive capacity of the lungs, whereas 8weeks does. We did not measure the lungs’ oxygen diffusive capacity, but we observed no significant changes in aerobic performance. The unchanged aerobic performance suggested that 4weeks of hypoxia at an average altitude of 4,900m might not have been sufficient stimulus for improving the lung’s oxygen diffusive capacity, but further research is needed to confirm this speculation.

We observed no significant changes in the parameters of aerobic performance, such as VO_2_max and maximal aerobic power, though a previous study described a decrease in VO_2_max after 8- to 10-week sojourns at >5,200m (Hoppeler et al., 1990a). Hypoxia has been shown to change muscle structure and so reduce sea-level aerobic performance. Hoppeler et al. (1990a) observed a 20% decrease in the cross-sectional area of muscle fiber after 8–10weeks of exposure above 5,200m. A similar dose of hypoxia also decreased mitochondrial volume density by 20% by deactivating mitochondrial biogenesis (Hoppeler et al., 1990b; Levett et al., 2012). HIF-1, which is induced by hypoxia and by an increase in free radicals (Askew, 2002), initiates mechanisms that decrease the quantity of free radicals in the mitochondria. The mechanisms induced by HIF-1 include more effective oxidative phosphorylation (Fukuda et al., 2007), lower pyruvate flux to mitochondria (Kim et al., 2006), the inhibition of mitochondrial biogenesis (Zhang et al., 2007), and greater mitochondrial autophagy (Zhang et al., 2008). All these mechanisms reduce oxidative stress together with the apoptosis of muscle fibers (Askew, 2002). Compensating for the negative effects of hypoxia by HIF-1, however, is limited by the dose of hypoxia. The limit seems to be crossed during a prolonged 8- to 10-week sojourn at altitudes above 5,200m as such hypoxic dose provoked 20% decrease in the cross-sectional area of muscle fiber (Hoppeler et al., 1990a) and 20% decrease in mitochondrial volume density (Hoppeler et al., 1990b; Levett et al., 2012). The 4weeks of hypoxia that the mountaineers in our study experienced were shorter, and the average altitude of 4,900m was lower than the 8- to 10-week sojourn and altitude of 5,200m that the mountaineers in a study of Hoppeler et al. (1990a) underwent. Given the insignificant changes in body weight and fat-free mass observed in our study, the dose of hypoxia that our alpinists experienced was probably too low to provoke a significant deterioration in their muscle structure, which in turn might explain the insignificant changes in their aerobic parameters. Brocherie et al. (2018) reported that repeated maximal-intensity hypoxic exercise superimposed to chronic hypoxic exposure reactivated HIF-1 and subsequent molecular downstream pathways. The hypothesis that higher climbing intensity at high altitude might improve adaptation and help to counteract muscle wasting needs further research. Unfortunately, we did not measure the level of climbing intensity in our study.

We demonstrated that 1month at a very high altitude averaging 4,900m significantly decreased the anaerobic threshold at sea level of the mountaineers we examined. An analysis of enzymatic activity in skeletal muscle biopsies of alpinists after an 8- to 10-week sojourn at extreme altitudes above 5,200m revealed a 25% decrease in their aerobic potential (Hoppeler et al., 1990a). Howald et al. (1990) observed a shift from aerobic toward anaerobic metabolism after 6weeks at extreme altitudes above 5,300m. HIF-1 increases anaerobic metabolism to compensate for the reduction in available energy from aerobic processes by activating the genes for glycolytic enzymes, such as lactate dehydrogenase, phosphofructokinase, glucose transporters, and lactate transporters (Cerretelli et al., 2009). The significant reduction in anaerobic threshold that we observed after 1month at the average very high altitude of 4,900m might be explained by a shift in skeletal muscle metabolism toward anaerobic processes.

Any increased work by the respiratory muscles and the related redistribution of blood would lead to an increase in the efficiency and strength of these muscles, which might cause the greater maximum tidal volume that we observed in our study. We found no data in the literature on changes in the structure of respiratory muscles at the tissue and cellular levels after a period of chronic hypoxia, but the greater availability of oxygen, energy, and building substrates for respiratory muscles at high altitude compared with locomotory muscles might well cause different changes in their structures. This topic needs further research.

### Anaerobic Performance

Our study concurred with others in finding that total anaerobic work and maximal anaerobic power decreased after 1month at altitudes above 3,500m (average 4,900m). Doria et al. (2020) observed a reduction in the mechanical and the metabolic parameters of the Wingate test after a 43-day expedition in the Himalayas (23days above 5,000m). Cerretelli and di Prampero (1985) reported a decrease in maximal anaerobic power after 5weeks of exposure to an altitude of 5,000m. Their study found no changes in the parameters of anaerobic performance after 3weeks at 5,000m, suggesting that changes in anaerobic performance depend on the duration of hypoxia.

The reduction in the anaerobic threshold that we observed might suggest an increase in anaerobic potential, especially the glycolytic potential in the skeletal muscles, which should cause an improvement of anaerobic parameters rather than deterioration. The discrepancy we observed between decreased total anaerobic work and maximal anaerobic power and the reduction in anaerobic threshold might be explained by a hypothesis holding that changes in the level of anaerobic threshold indicate only a direction of metabolic changes in the skeletal muscle. The predominance of anaerobic processes does not necessarily indicate higher levels and greater activity of the glycolytic enzymes, but might result from less deterioration in anaerobic metabolism than in aerobic metabolism.

Hypoxia can reduce the sensitivity of skeletal muscles’ cell membranes because of a reduction in the quantity and the activity of sodium-potassium adenosine triphosphatase (Na^+^/K^+^ ATPase), which might lead to more rapid fatigue during exercise (Aughey et al., 2005). The lower quantity and activity of Na^+^/K^+^ ATPase results in a deterioration of the cell membrane’s sodium and potassium exchange, which would reduce impulse transmission along the muscle fibers and consequently slow the generation and transmission of impulses through the nerve fibers. Furthermore, a lower quantity and activity of Na^+^/K^+^ ATPase might provoke a slower impulse generation frequency in the motoneurons and reduce the fast-twitch (FT) muscle fibers’ stimulation, which would likely decrease the power of the contractions they generate. This hypothesis would explain the reduced anaerobic performance observed in our study.

The average rate of ascent among climbers requires about 50% of VO_2_max (West et al., 2007). Physical activity in hypoxic conditions seems to be naturally regulated and kept at the most effective level given the metabolic possibilities of the muscles. The low climbing intensity might increase the number of motoneurons of slow-twitch (ST) muscle fibers that are engaged and decrease the quantity of the engaged motoneurons of FT fibers. The reduction in the number of FT fibers recruited at altitude might result in the decrease in anaerobic performance at sea level we observed in alpinists returning from a high-altitude expedition. The lower contraction power after a high-altitude sojourn would explain the reduced maximal anaerobic power and the total anaerobic work we observed in the Wingate test of our study.

### Anthropometric Data

Contrary to the results of other authors (Consolazio et al., 1968; Boyer and Blume, 1984; Macdonald et al., 2009), our study did not show a significant decrease in body weight after a sojourn at high altitude. The significant reduction in body weight that others have observed came after 2weeks of exposure to an altitude below 5,400m (Boyer and Blume, 1984) and to 28days at an altitude of 4,300m (Consolazio et al., 1968). Macdonald et al. (2009) observed a significant body weight loss of 2.4kg after 3weeks of trekking in the Himalayas. Our results can be explained by recovery of any lost body weight in the 7days between the exposure to altitude and the measurements we completed at sea level. The Operation Everest III study (Westerterp-Plantenga et al., 1999) was performed in simulated extreme hypobaric conditions and reported that 63% of lost body weight was recovered within 4days of returning to sea level, which was explained by a physiological retention of fluids. Body weight also recovered after 1week of rest at sea level after a 1-month sojourn at 4,300m (Consolazio et al., 1968). Less body weight will be lost with an appropriate increase in the energetic value of the food consumed at altitude to cover the increased energy demand (Butterfield et al., 1992). We did not analyze the diet followed by the alpinists in our study, but the fact that body weight did not change suggested that their energy intake was adequate to meet their energy expenditure.

Fat mass was the only component of body composition to change significantly in our study. The reduction in fat mass from 14% of body weight before the expeditions to 11.5% afterward accords with other studies that report alterations in body composition after sojourns at high altitudes below 5,400m. Boyer and Blume (1984) reported that fat mass is the main component of body composition responsible for total body weight reduction in sojourns below 5,400m. Macdonald et al. (2009) reported a decrease in total body weight after 21days of trekking in the Himalayas, that loss composed 45% fat, 20% residual mass (principally protein and glycogen), and 35% of total body water.

How prolonged exposure to extreme altitudes above 5,000m affect body composition remains unclear. Boyer and Blume (1984) found that a prolonged sojourn above 5,400m reduced body weight: 73% to lost proteins and only 27% to a reduction in fat mass. In contrast, Reynolds et al. (1999) reported that reduced fat mass was the main change in the body composition of alpinists on an Everest expedition who spent 9weeks above 5,300m. Westerterp et al. (1994) observed that a 21-day sojourn at an altitude above 6,500m reduced body weight, of which 70% was due to reduced fat mass. Reynolds et al. (1999) suggested that humans use fat reserves as an energetic substrate to cover the increased energetic demands at altitude and so spare losing muscle mass. The results reported by Boyer and Blume (1984); however, do not confirm Reynolds et al.’s suggestion.

### Hematological Data

We observed no changes in the hematological parameters we examined. In contrast, Reynafarje et al. (1959) observed a 10% increase in RBC after 4weeks at 4,500m. Similarly, Ferretti et al. (1990a) found that a sojourn of 8–10weeks at altitudes above 5,200m caused a significant increase in Hb by 14.1% and Hct by 12.2%.

The 7days between the mountaineers’ descent from altitude and when we took their measurements might have affected our results, though Reynafarje et al. (1959) found that higher levels of RBC are maintained for 2–4weeks after descent. Rice et al. (2001) observed neocytolysis-hemolysis of young circulating red blood cells (neocytes) in nine high-altitude residents with polycythemia after they descended from 4,380m to sea level (Rice et al., 2001). The level of their RBC decreased by 7% in their first few days at lower altitude, a phenomenon that might explain the absence of changes in the hematological parameters we examined.

The unchanged hematological parameters that we observed might be partly explained by the fact that each individuals’ hematopoietic system reacts differently to hypoxia: Some respond to hypoxia with an increase in their erythropoietin levels and a subsequent increase in Hb and Hct; others do not show this reaction (Chapman et al., 1998). Our alpinists might have had hematopoietic systems that did not respond to the hypoxic stimulus that was delivered.

The unchanged Hb concentration we observed might also result from an increase in red cell mass and plasma volume in a similar ratio after a month at very high altitude.

### Strengths and Limitations

Our study is one of very few that analyze aerobic and anaerobic performance, body mass and composition, and hematological parameters before and after a sojourn at very high altitude. Our results expand the current knowledge of how prolonged hypoxia at very high altitude affects sea-level physical performance. Given that very high altitudes of 3,500–5,500m include the most popular trekking and climbing areas, our work concerns a large group of people that includes mountaineers and high-altitude tourists.

Our post-expedition measurements were done within 7days after the mountaineers descended below 3,500m, which limits comparisons with other studies that differ in the number of days between the end of exposure to hypoxia and when sea-level measurements are taken. Mountaineers are usually examined days after they descend to sea level because they must travel from remote mountains to a suitable laboratory (Hoppeler et al., 1990a).

The reduction in anaerobic threshold we observed might result from our methodology. We determined anaerobic threshold using a non-invasive method based on the ventilatory threshold, which might not correlate with the anaerobic threshold after a high-altitude sojourn in the same way as before exposure. During adaptation to high altitude, the peripheral chemoreceptors are sensitized, resulting in them responding faster to hypoxia and hypercapnia (Dempsey and Forster, 1982; Schoene et al., 1984; Sato et al., 1992). Sensitization of the peripheral chemoreceptors at altitude might provoke a quicker ventilatory response to the lower levels of arterial carbon dioxide at sea level, which might reduce the ventilatory threshold at sea level after a sojourn at high altitude. HIF-1 causes the metabolism to shift toward higher utilization of carbohydrates as energy substrates and might also provoke a higher production of carbon dioxide during exercise at the same level of VO_2_max after descent to sea level. The surfeit of carbon dioxide would lead to faster ventilation and shift the ventilatory threshold to the left on the ventilation-power curve of the incremental exercise test to exhaustion.

### Conclusion

The dose of hypoxia experienced in 1-month mountaineering expeditions at very high altitude averaging 4,900m impairs anaerobic performance and the anaerobic threshold at sea level, has a neutral effect on aerobic performance and hematological parameters, and induces lower fat mass.

## Data Availability Statement

The raw data supporting the conclusions of this article will be made available by the authors, without undue reservation.

## Ethics Statement

The studies involving human participants were reviewed and approved by the Regional Ethics Committee of the Medical University of Gdańsk. The patients/participants provided their written informed consent to participate in this study.

## Author Contributions

RS and TG: conceptualization, formal analysis, and writing – original draft preparation. RS, TG, and EZ: methodology, validation, and data Curation. RS: investigation and supervision. RS and MS: resources and visualization. RS, TG, EZ, MS, and RL: writing – review and editing. All authors contributed to the article and approved the submitted version.

## Funding

This work was supported by the Medical University of Gdańsk (grant number 664/66-0621/66/282). Medical University of Gdańsk financed the language correction and open access publication fee.

## Conflict of Interest

The authors declare that the research was conducted in the absence of any commercial or financial relationships that could be construed as a potential conflict of interest.

## Publisher’s Note

All claims expressed in this article are solely those of the authors and do not necessarily represent those of their affiliated organizations, or those of the publisher, the editors and the reviewers. Any product that may be evaluated in this article, or claim that may be made by its manufacturer, is not guaranteed or endorsed by the publisher.
